# Supplementary material for: Chemogenomic analysis reveals key role for lysine acetylation in regulating Arc stability
Source: Nat Commun. 2017 Nov 21;8:1659. doi: 10.1038/s41467-017-01750-7 (PMC5698418; doi:10.1038/s41467-017-01750-7)
Supplement: Supplementary file 1 — Supplementary Information [file 41467_2017_1750_MOESM1_ESM.docx]

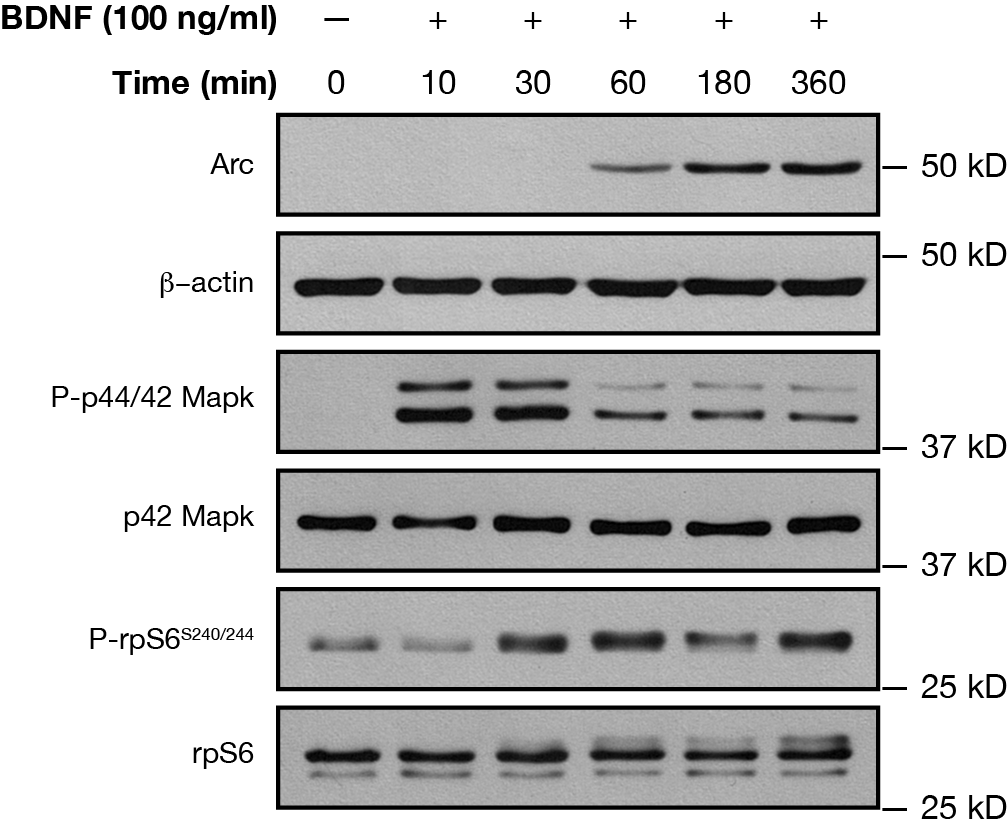


**Supplementary Fig. 1. Time-course of BDNF-induced Arc expression in mouse primary cortical neurons.** Dissociated DIV14 cortical neurons were treated with exogenous BDNF (100 ng ml^-1^) for the indicated time. Lysates were analyzed by western blot for Arc, phospho-p44/42 Mapk, and phospho-rpS6 to reveal the time-dependent profile of each antigen. β-actin, p42 Mapk, and rpS6 were used as loading controls.

**
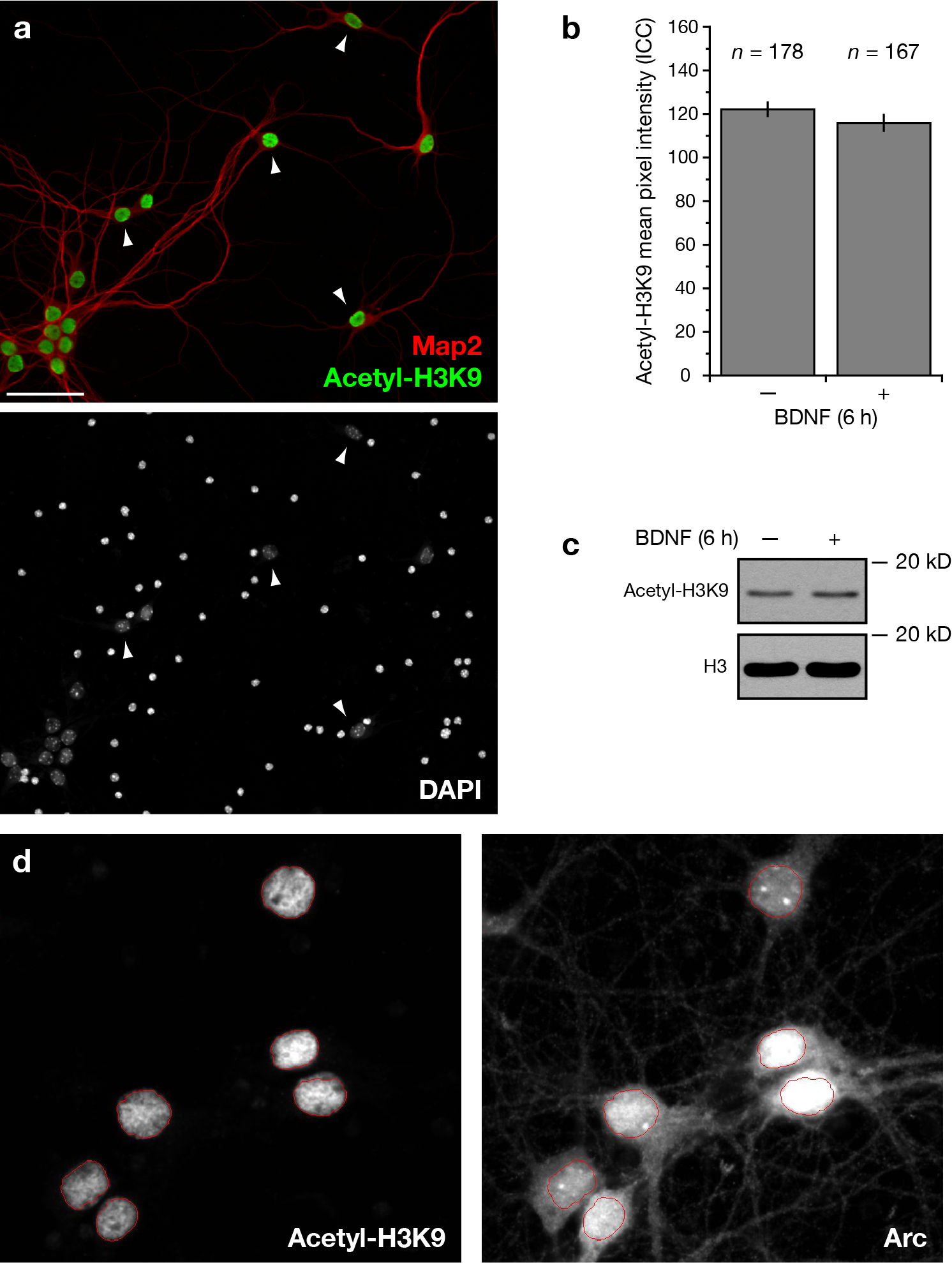
**

**Supplementary Fig. 2.** Characterization of acetyl-H3K9 as a primary cortical neuron nuclear marker. **a** Top panel shows representative co-immunostaining of acetyl-H3K9 (green fluorophore) and MAP2 (red fluorophore) in mouse primary cortical neurons. Strong acetyl-H3K9 immunostaining is restricted to MAP2-positive cells (neurons) and not found in smaller nuclei corresponding to non-neuronal cells, as seen in lower panel with DAPI staining. Arrowheads indicate the exact same position in each panel. *Scale bar* 50µm. **b** Quantification show that acetyl-H3K9 immunofluorescence intensity levels in primary cortical neurons are unaffected by application of BDNF for 6 h. **c** Western blot analysis confirms the absence of change in acetyl-H3K9 level in response to BDNF. **d** Acetyl-H3K9 immunostaining was used as a mask for the automated analysis of nuclear Arc expression in cortical neurons for our chemogenomic screen.

**
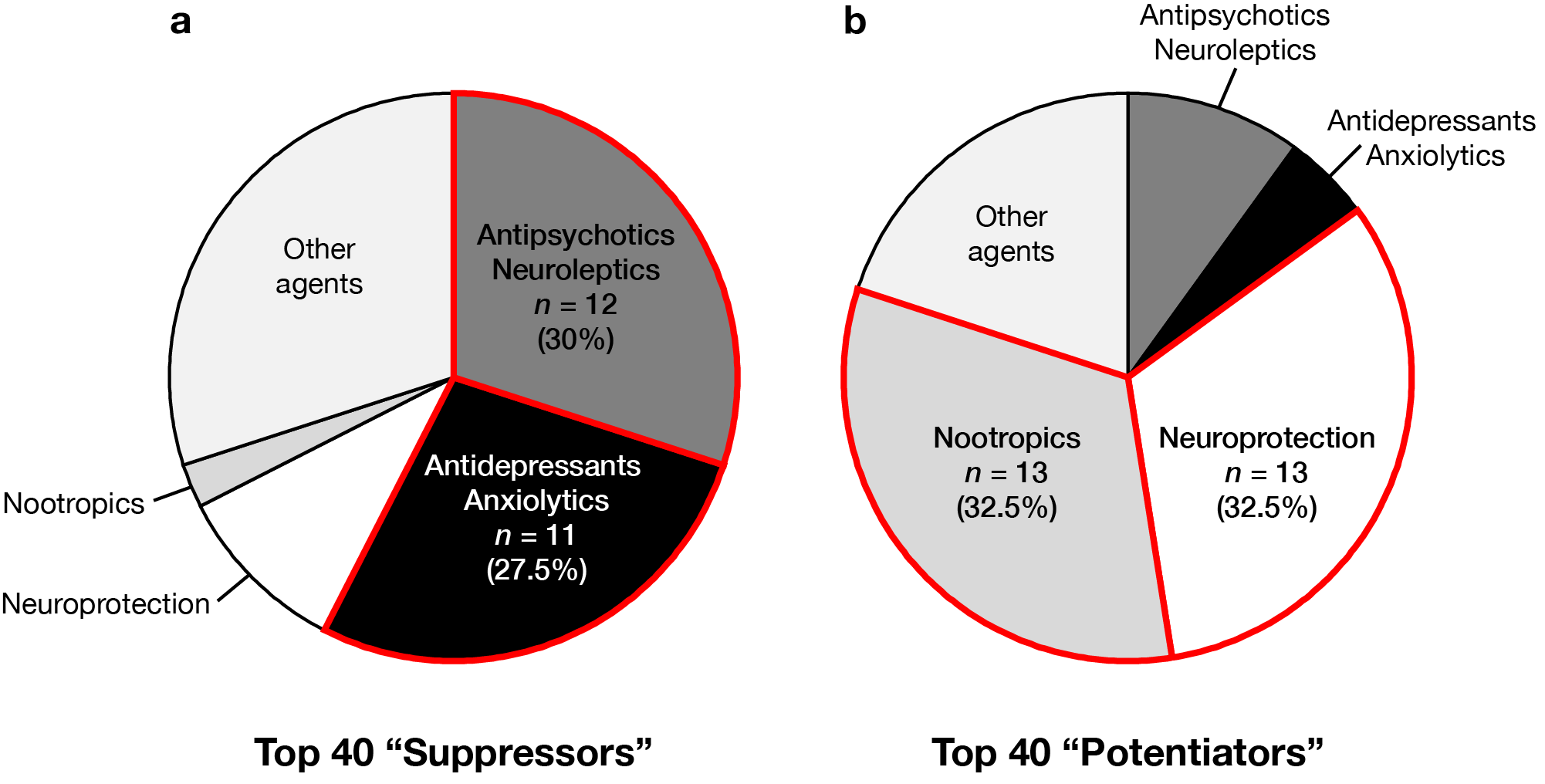
**

**Supplementary Fig. 3.** Grouping based on therapeutic qualities of compounds found to affect BDNF-induced Arc expression in primary cortical neurons. **a-b** Survey of the 40 most potent agents at each extremity of our chemogenomic screen dataset reveals that the majority of the considered “suppressors” were drugs with antipsychotic or antidepressant activity (**a**), while the majority of the “potentiators” had neuroprotective and/or nootropic qualities (**b**).

**
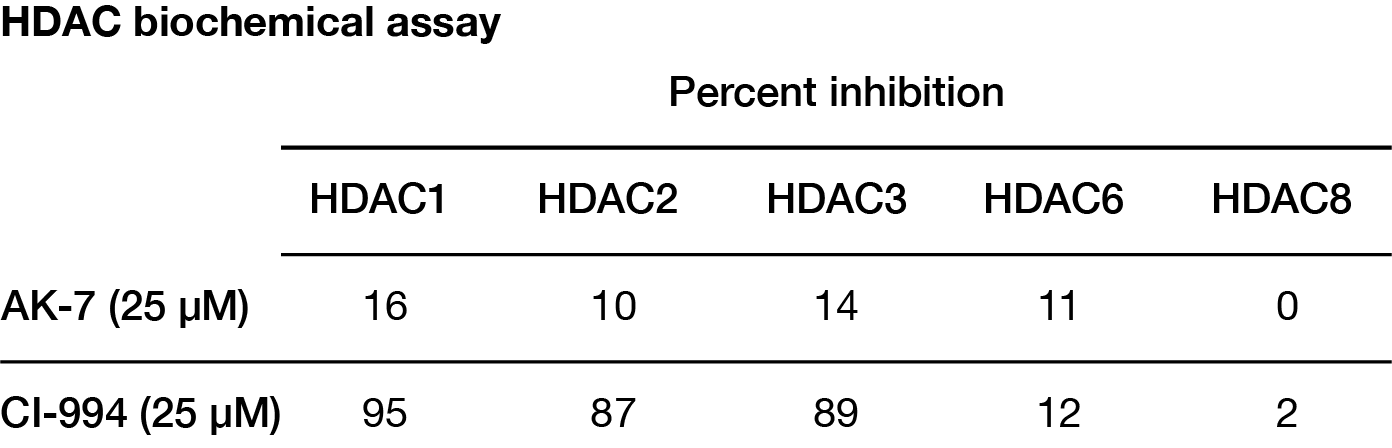
**

**Supplementary Fig. 4.** AK-7 and CI-994 inhibitory activity of HDACs. The inhibitory activity of AK-7 and CI-994 toward representative class I (1/2/3), IIA (6), and class IIB (8) HDACs at a 25 µM concentration was assessed by BPS Bioscience with their *in vitro* biochemical assay.

**
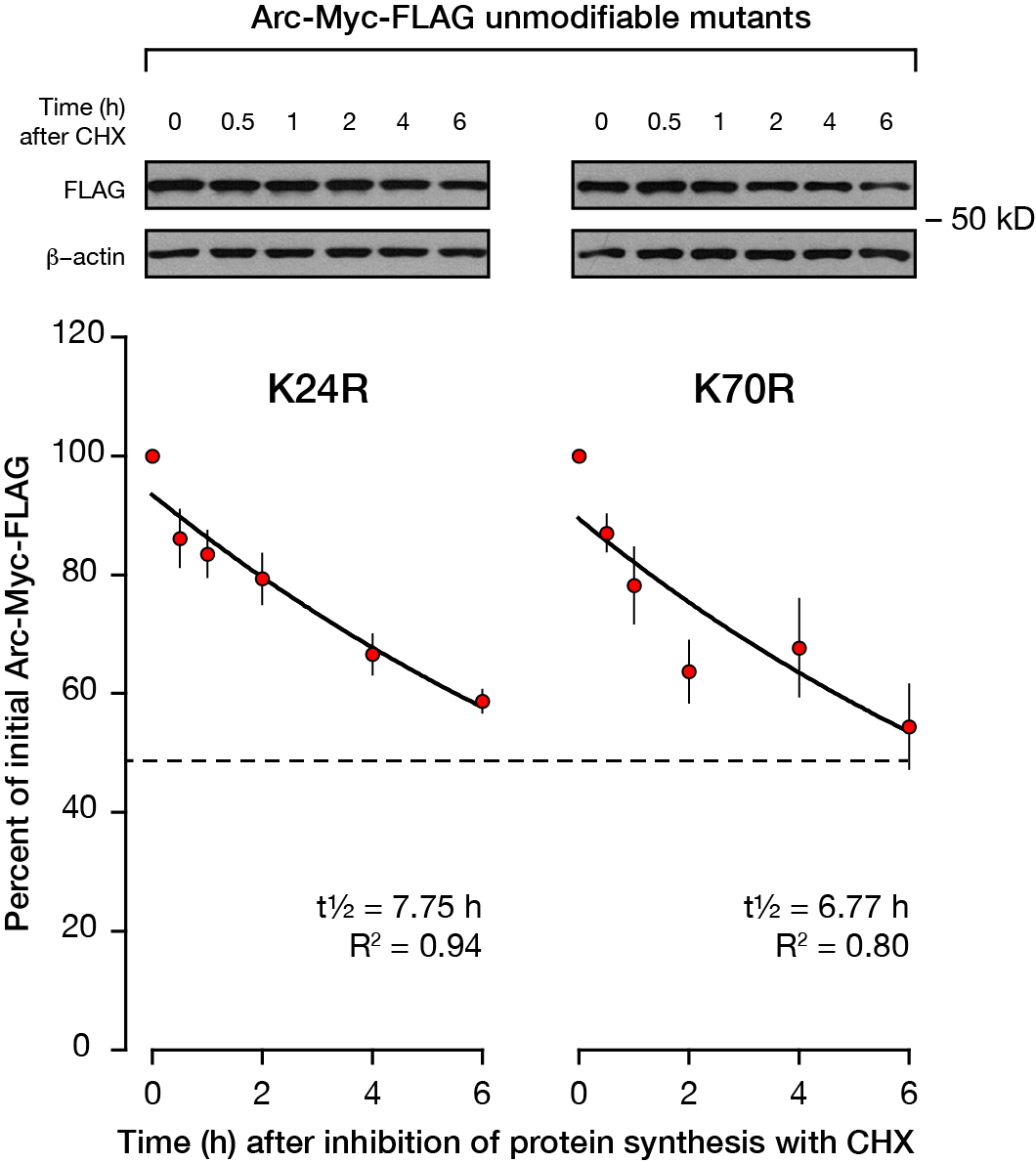
**

**Supplementary Fig. 5.** Protein stability of Arc-Myc-FLAG K24R and K70R mutants. Mouse non-acetyl/unmodifiable mutant Arc-myc-FLAG proteins (K24R and K70R) were overexpressed in different Neuro2a cultures and analyzed as for assays conducted with WT and acetyl-mimic/unmodifiable mutants. Each data point is represented as mean (± SEM) calculated from three biological replicates and is presented as percentage of Arc abundance at the initial time-point after CHX application (*t* = 0). Arc protein half-life was estimated by exponential decay curve fit and representative western blots are presented on top.

**
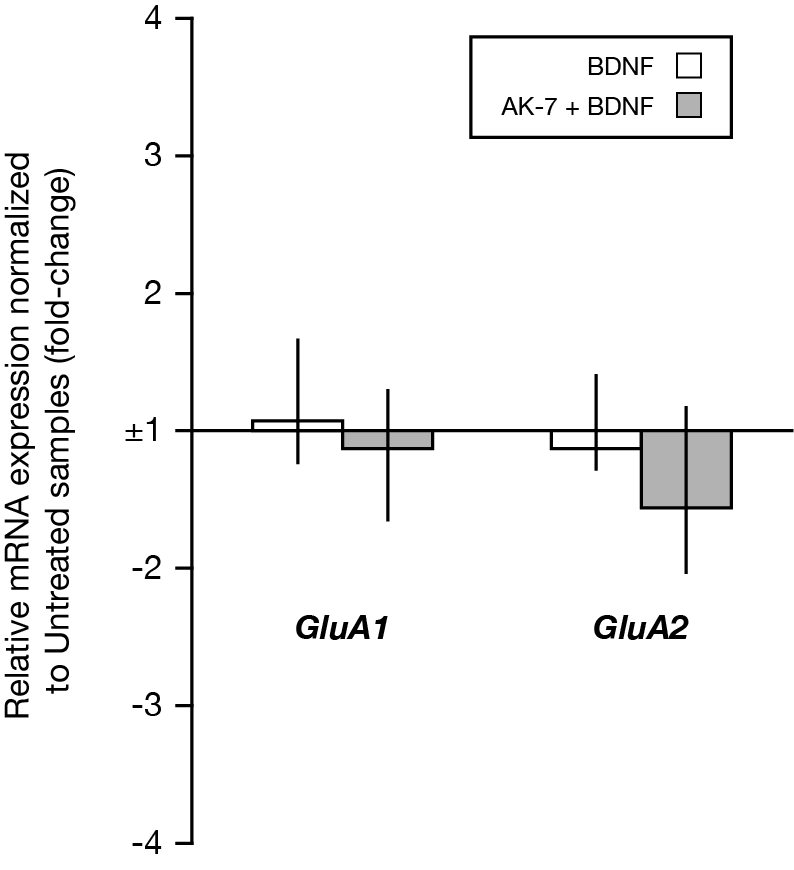
**

**Supplementary Fig. 6.** AK-7 does not affect *GluA1 and GluA2* mRNA expression. *GluA1 and GluA2* mRNA expression in DIV14 cortical neurons treated for 6 h with BDNF plus vehicle or BDNF plus KDAC inhibitor AK-7 (16.7µM) was assessed by RT-qPCR. Bars represent mean fold-change and error bars indicate range from five biological replicates.

**
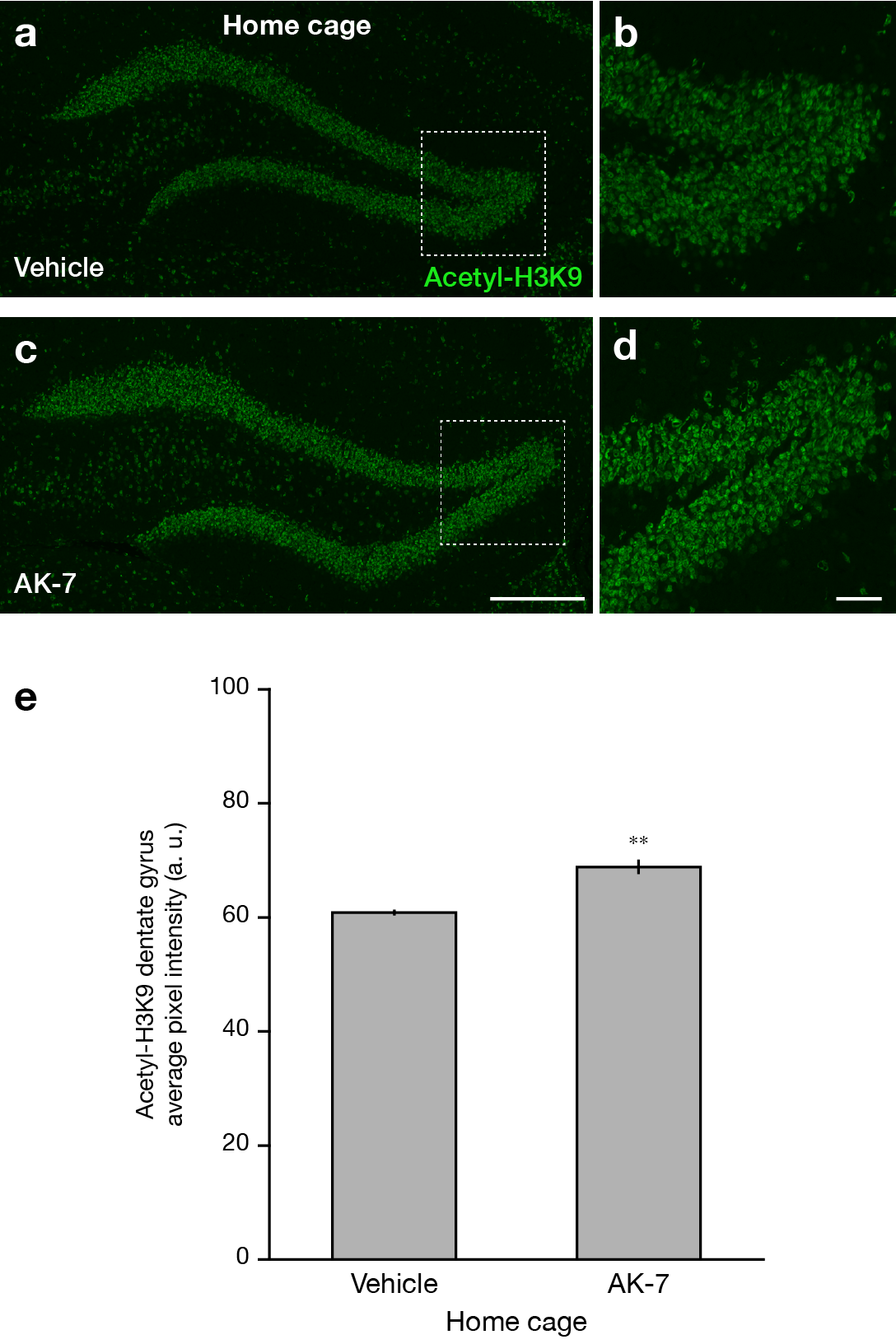
**

**Supplementary Fig. 7.** KDAC inhibitor AK-7 increases H3K9 acetylation *in vivo*. **a-d** Representative captures of acetyl-H3K9 immunostaining in the dentate gyrus of mice 3h after intraperitoneal injection of vehicle (**a-b**) or AK-7 (**c-d**, 30 mg kg^-1^). Mice were returned to their home cage immediately after the injection and left undisturbed for the time-period before sacrifice. *Scale bar* 250µm (**a** and **c**). High-magnification image of the area highlighted by a dotted square in **a** and **c** are presented in **b** and **d**, respectively. *Scale bar* 50µm. **e** Quantification of acetyl-H3K9 immunofluorescence for the whole dentate gyrus of mice injected with vehicle or AK-7. Data represents average for three independent biological replicates for each experimental condition. ***p* < 0.01, two-tailed *t*-test.

**
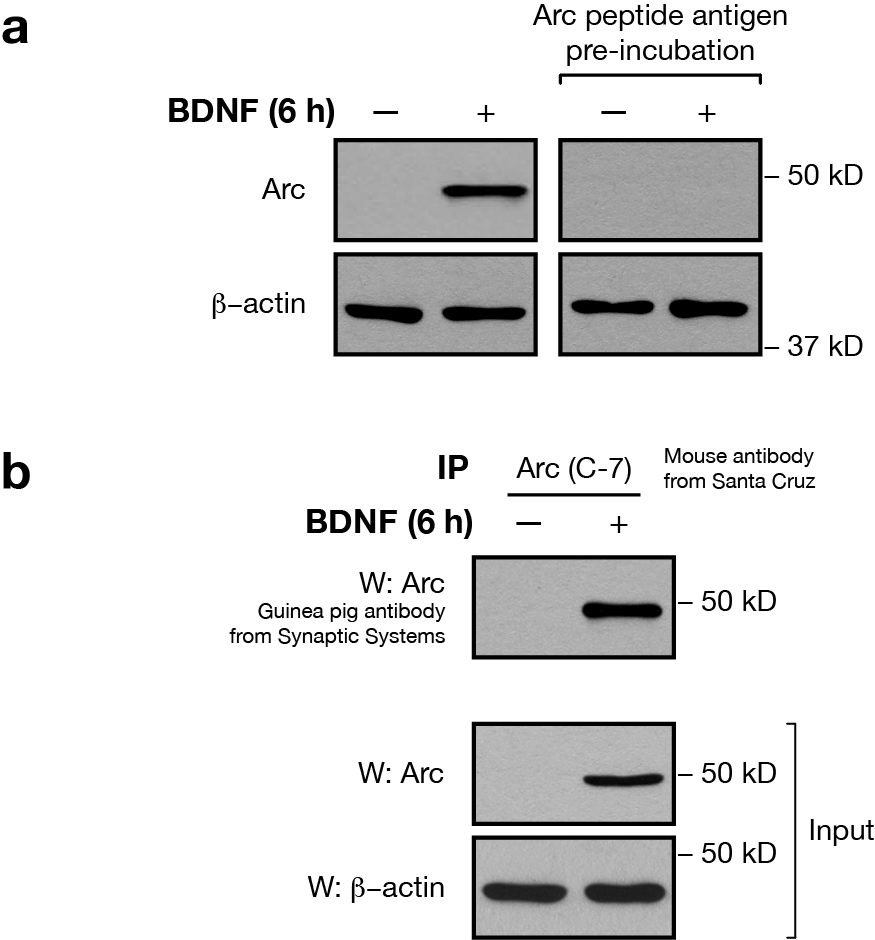
**

**Supplementary Fig. 8.** Validation of Arc antibodies antigen specificity. **a** Lysates from primary cortical neurons treated with BDNF for 6 h to induce Arc expression were analyzed by western blot with the Synaptic Systems anti-Arc antibody (left). Specificity of the antibody was assessed by pre-absorbing the anti-Arc antibody with 5 µg ml^-1^ of full-length recombinant Arc protein that has been used for immunization (Synaptic Systems, #156-0P). **b** The specificity of the two anti-Arc antibodies used in our study was also verified by immunopurifying Arc from BDNF-treated primary cortical neurons with the Santa Cruz anti-Arc (C-7) antibody and performing western blotting with the Synaptic Systems antibody.

**
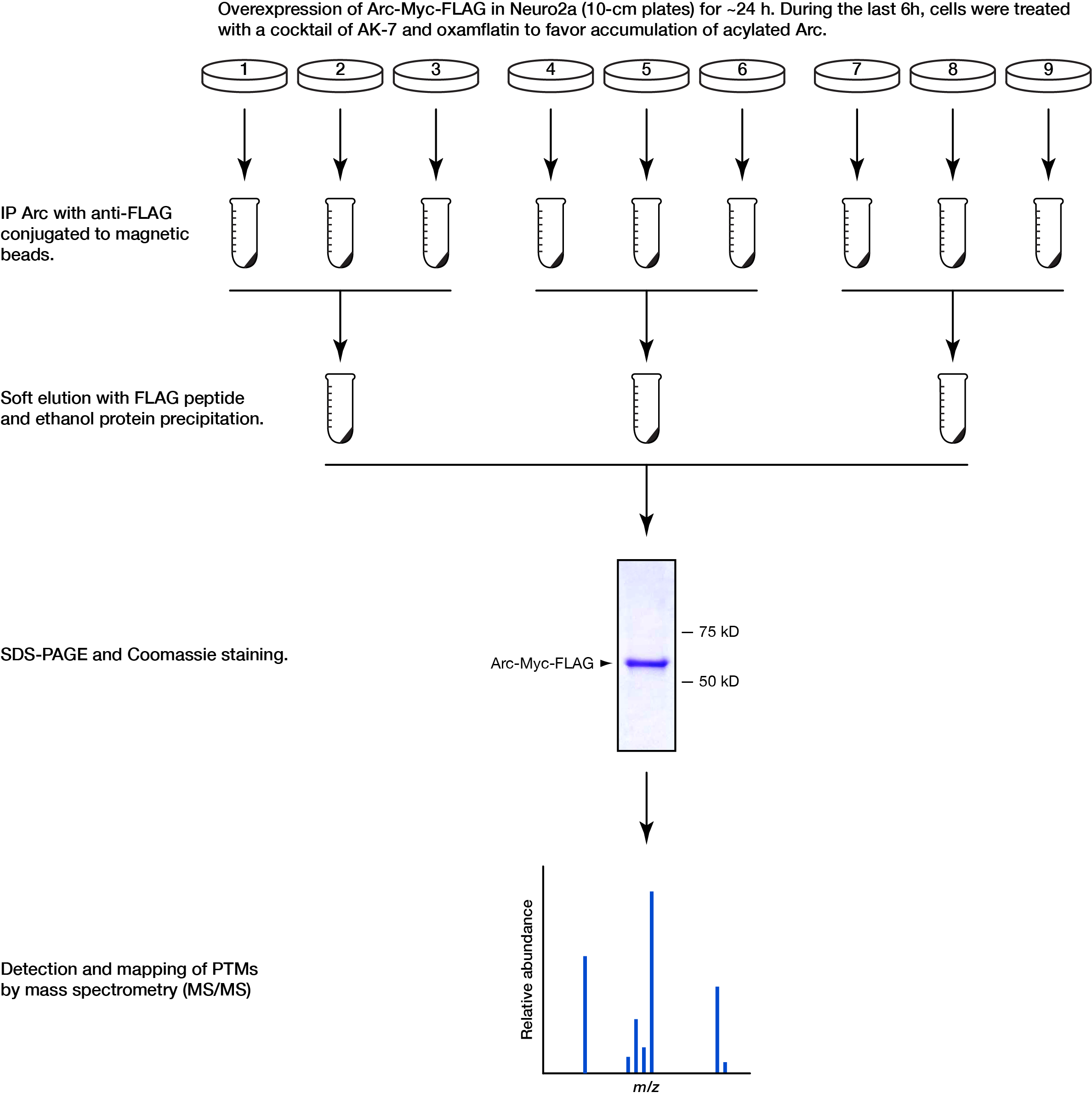
**

**Supplementary Fig. 9.** Summary of experimental strategy adopted to collect sufficient amount of immunopurified Arc-Myc-FLAG protein for mass spectrometry analysis. Arc-Myc-FLAG overexpressed in Neuro2a cells treated for 6 h prior to cell lysis with the KDACs inhibitors oxamflatin and AK-7 was immunopurified from nine separate cell lysates (10-cm plate cultures) with anti-FLAG M2 antibody conjugated to magnetic beads. Arc protein was eluted from antibody with FLAG peptide and then concentrated by ethanol precipitation. Finally, the separate samples were pooled together and the single Coomassie stained Arc-Myc-FLAG band shown was excised and in-gel digested using trypsin prior to mass spectrometric analysis.

**
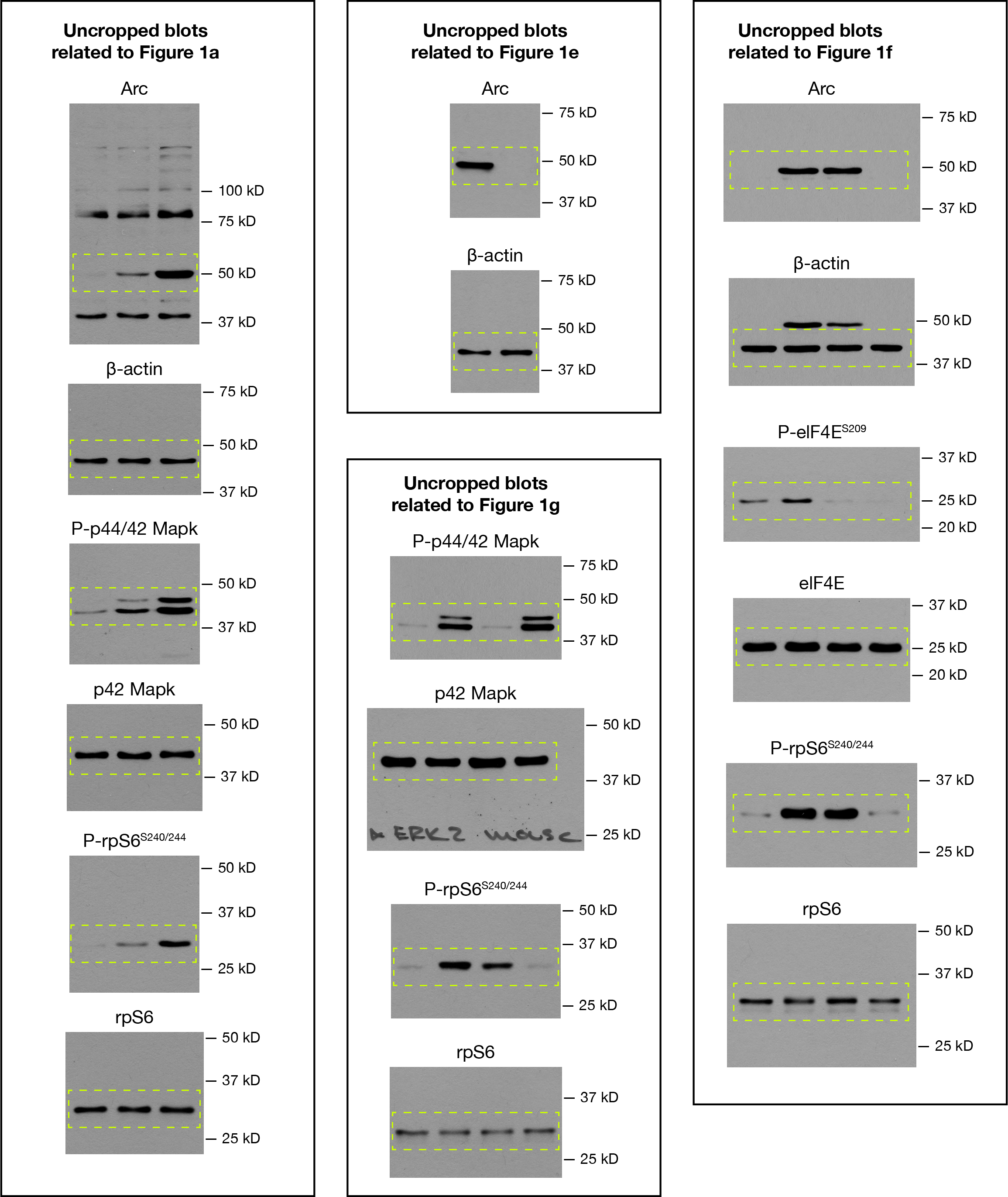
**

**Supplementary Fig. 10**

**
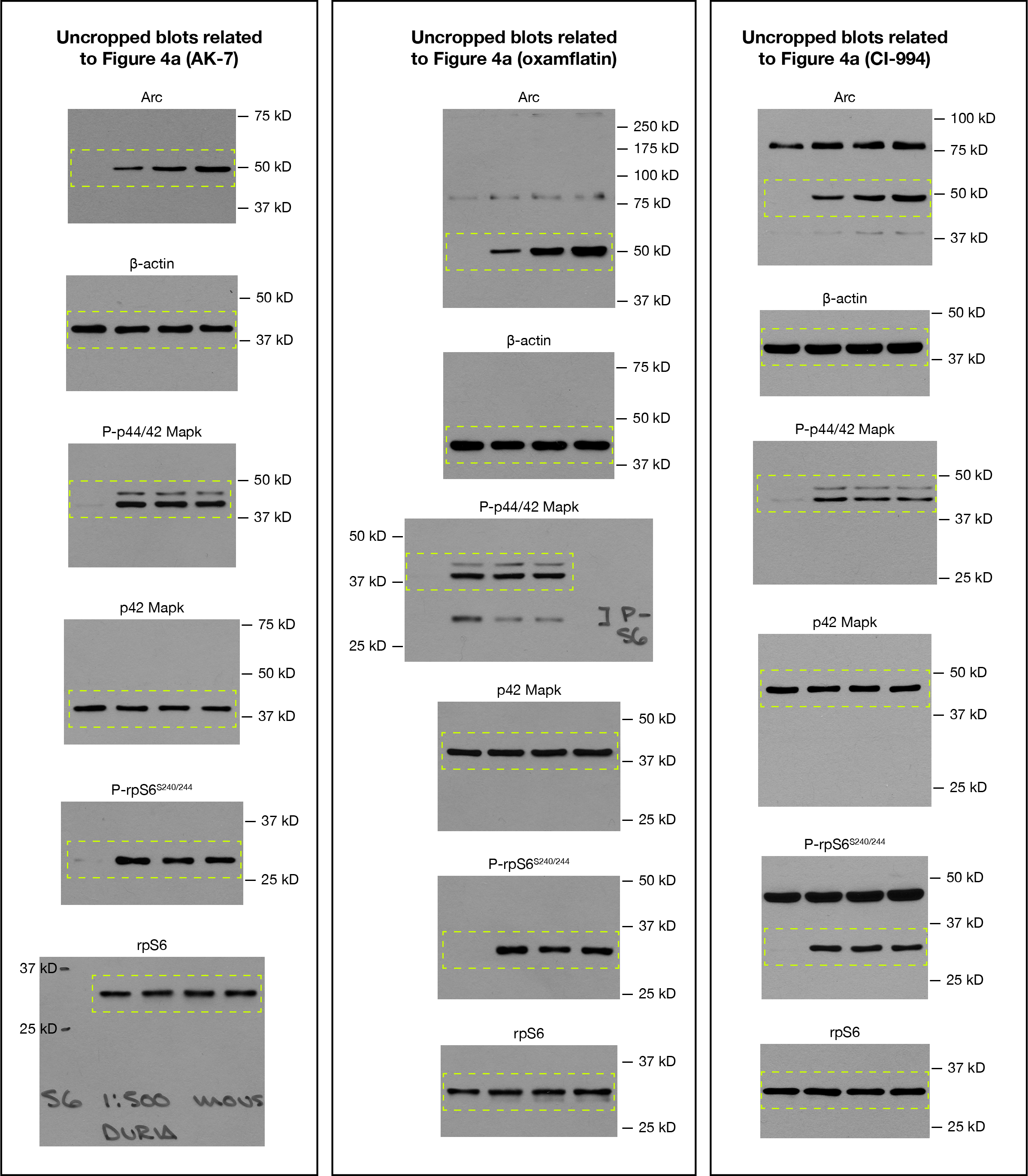
**

**Supplementary Fig. 11**

**
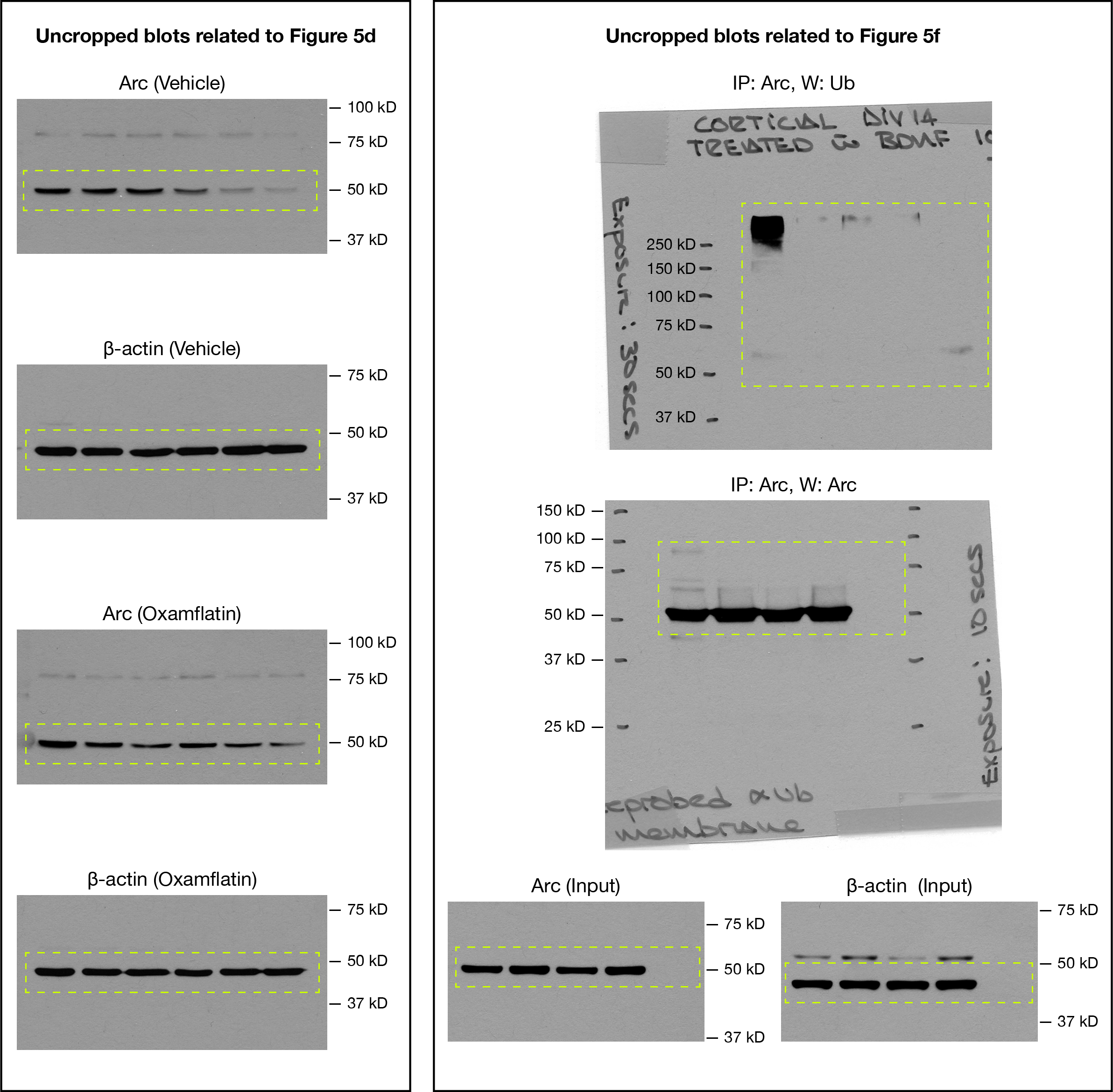
**

**Supplementary Fig. 12**

**
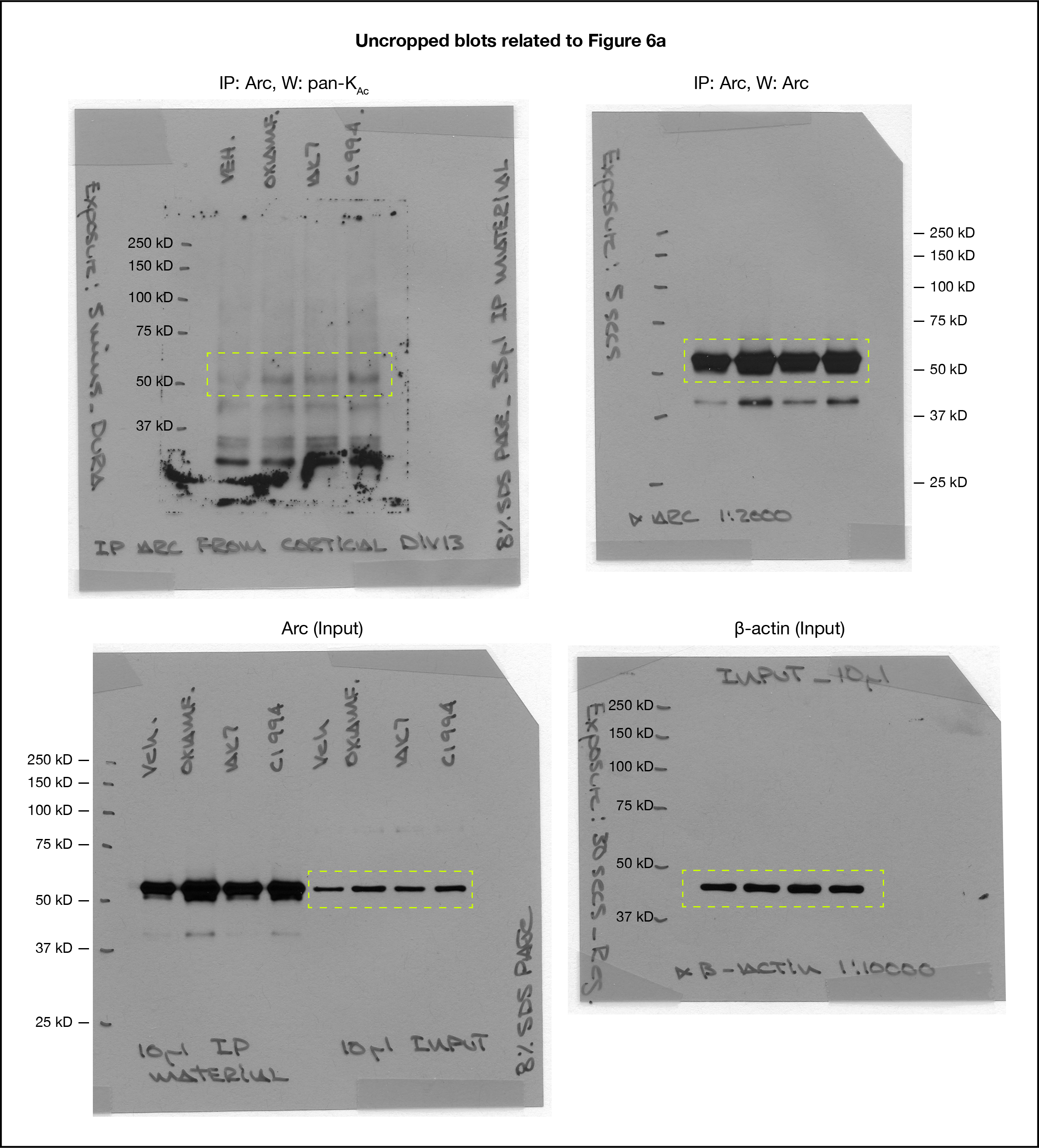
**

**Supplementary Fig. 13**

**
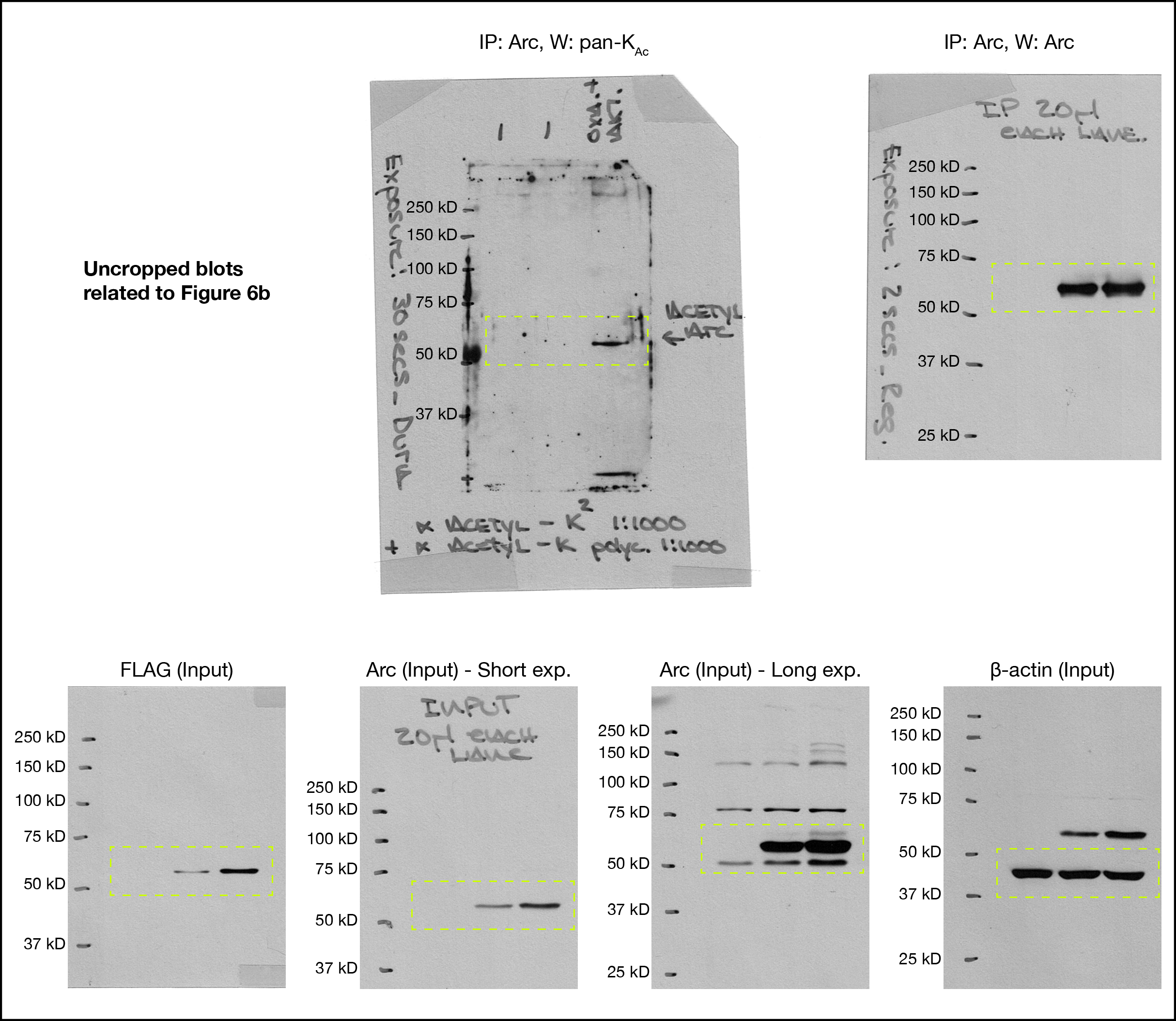
**

**Supplementary Fig. 14**

**
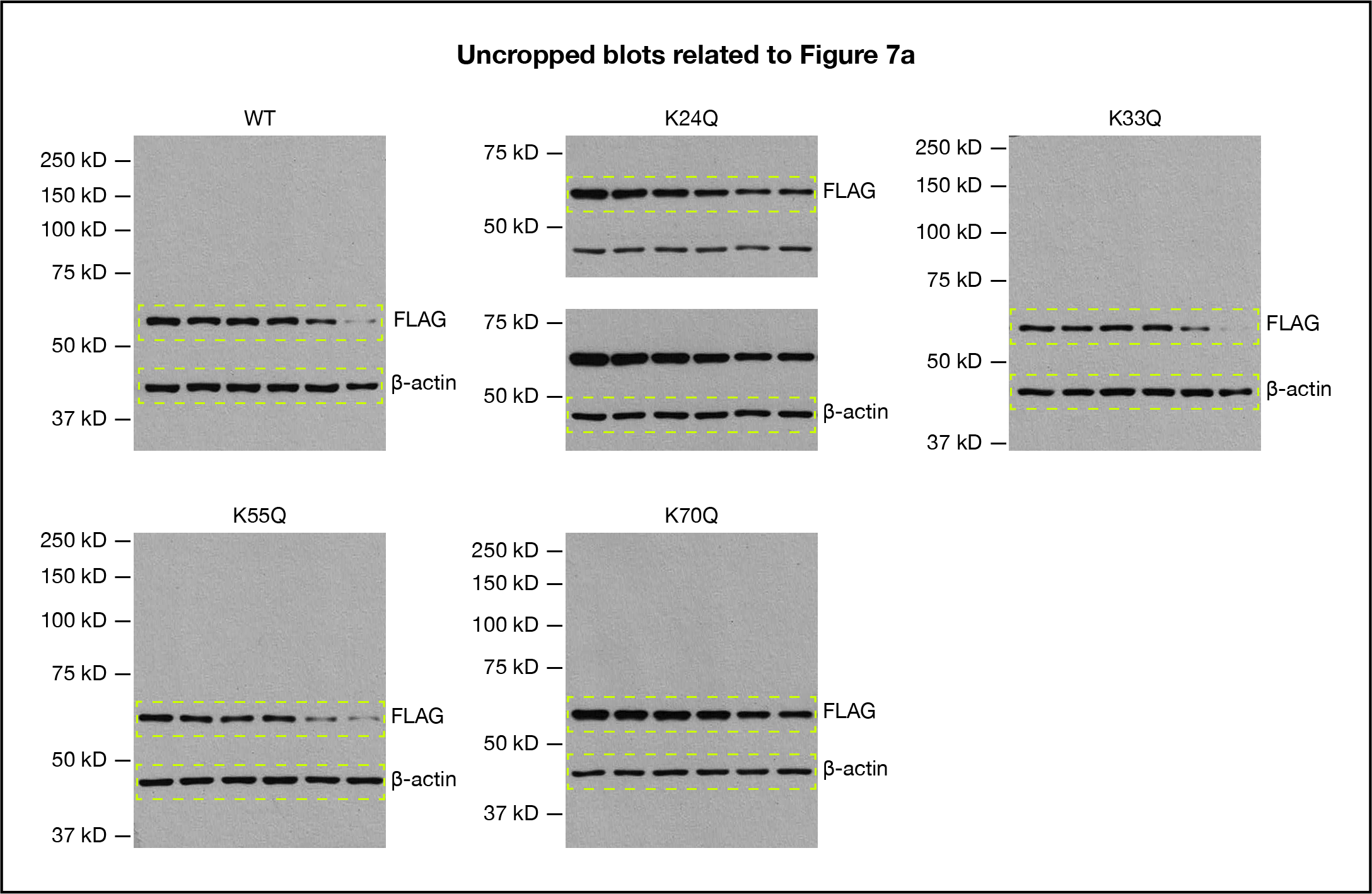
**

**Supplementary Fig. 15**


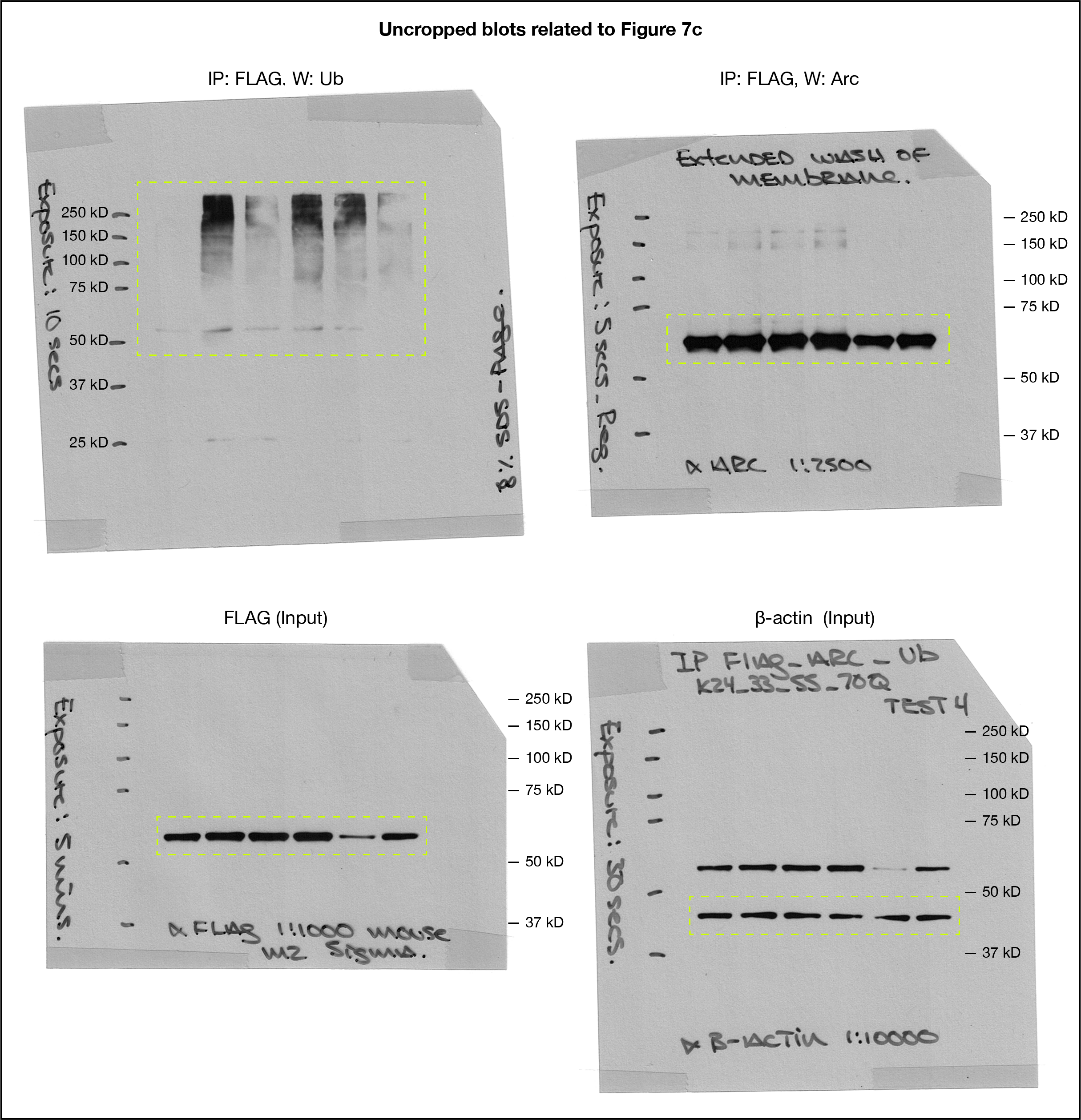


**Supplementary Fig. 16**

**
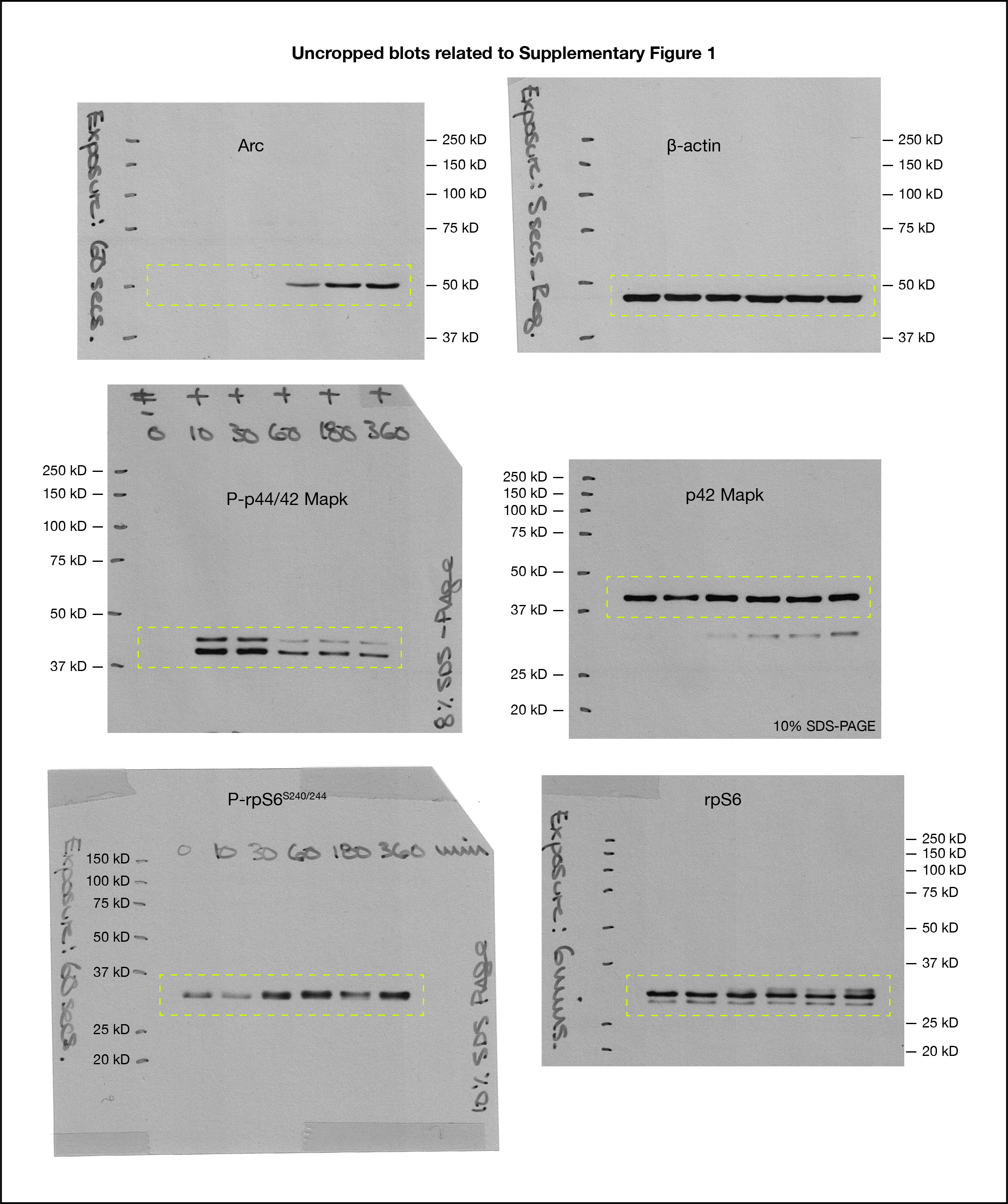
**

**Supplementary Fig. 17**

**
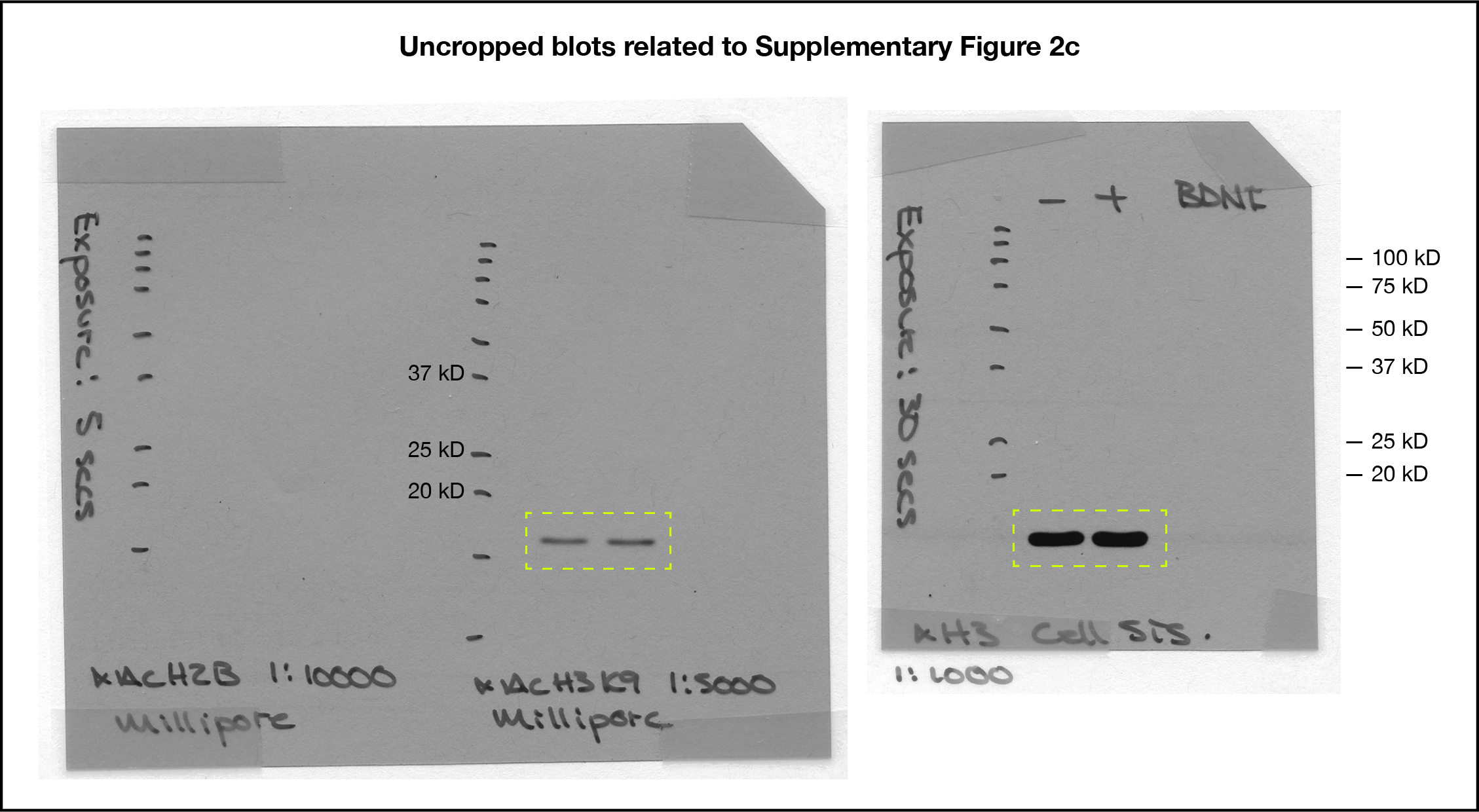
**

**Supplementary Fig. 18**

**
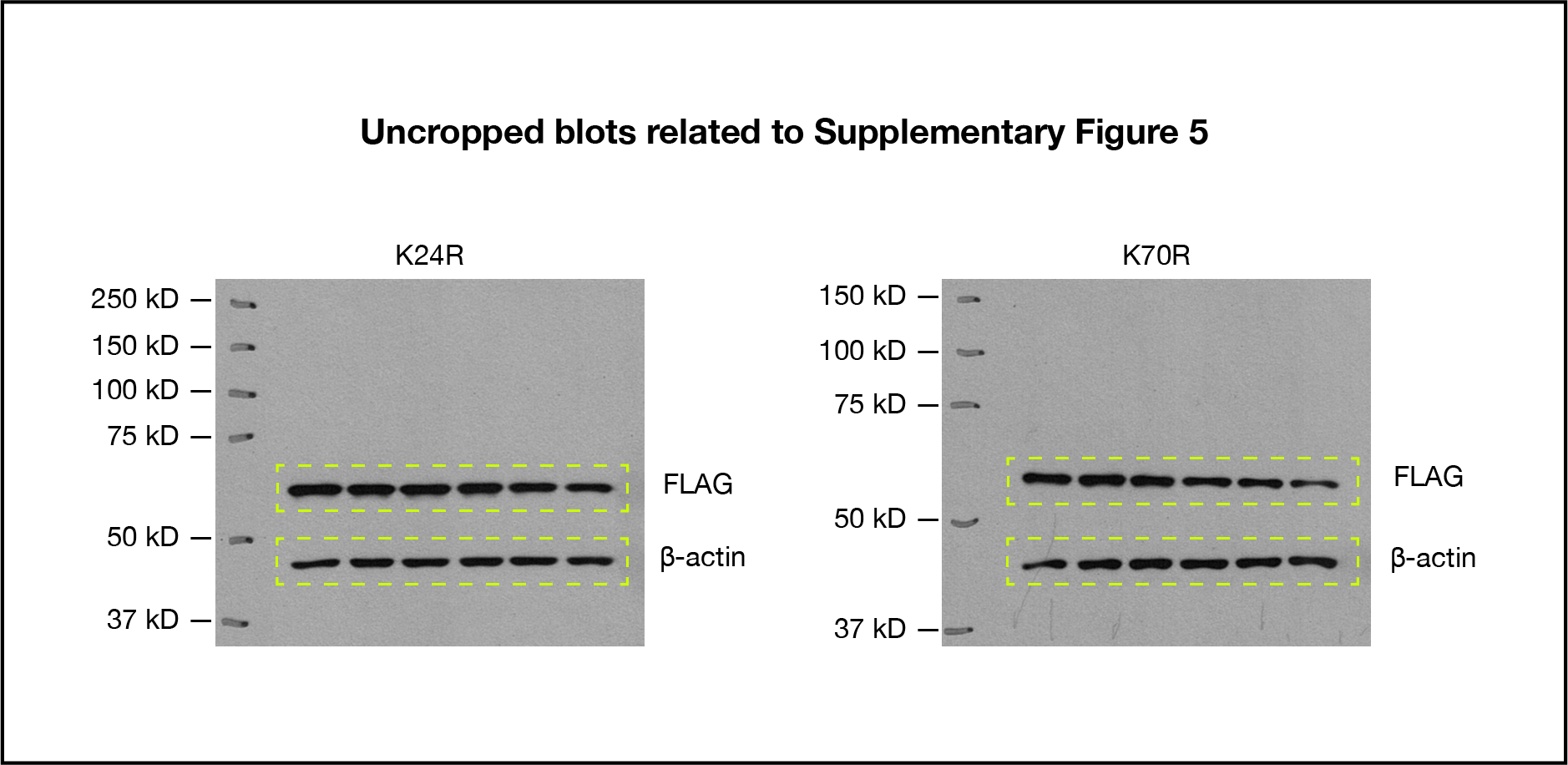
**

**Supplementary Fig. 19**

**
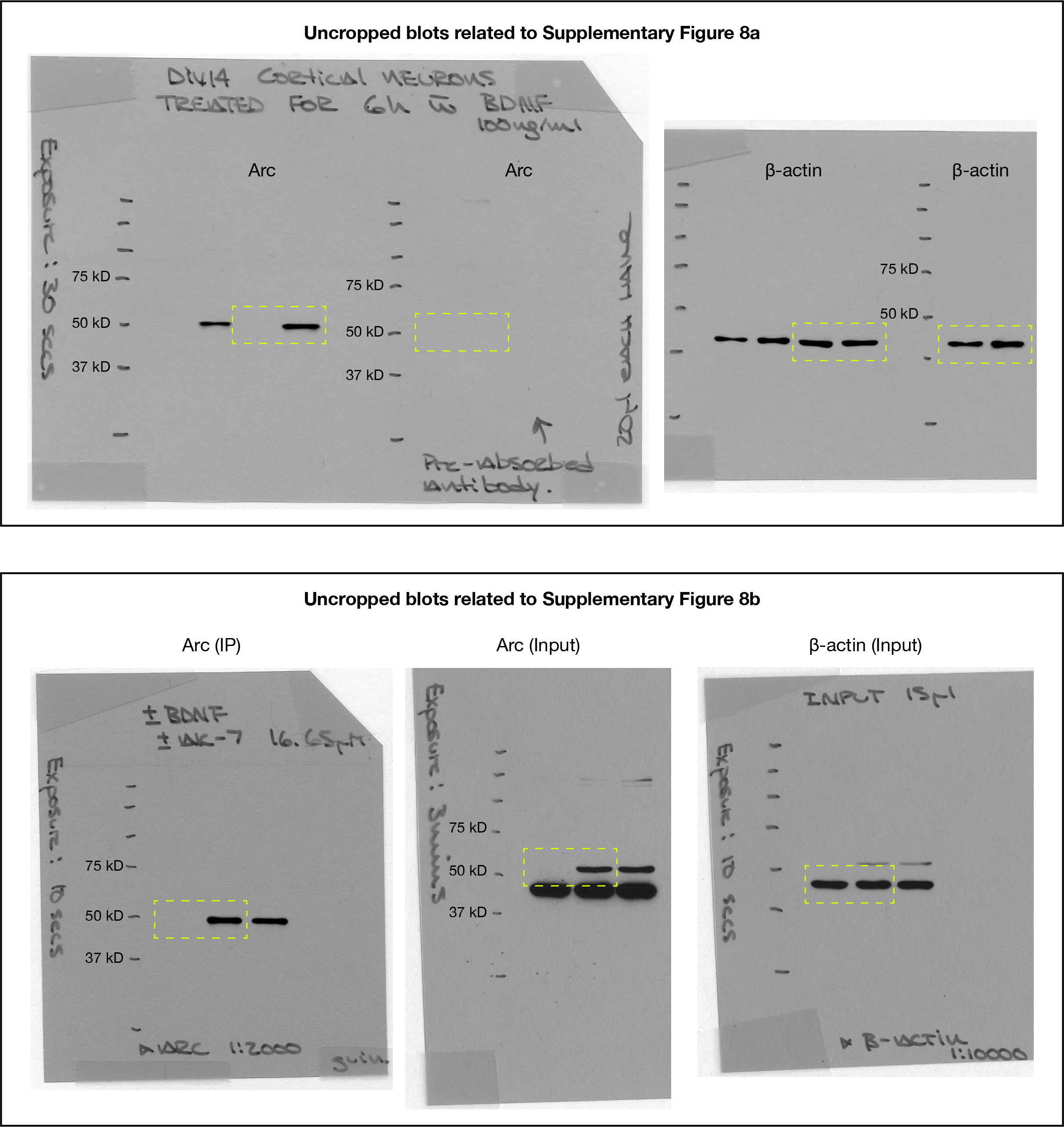
**

**Supplementary Fig. 20**
