# Supplementary material for: Chemogenomic analysis reveals key role for lysine acetylation in regulating Arc stability
Source: Nat Commun. 2017 Nov 21;8:1659. doi: 10.1038/s41467-017-01750-7 (PMC5698418; doi:10.1038/s41467-017-01750-7)
Supplement: Supplementary file 3 — Descriptions of Additional Supplementary Files [file 41467_2017_1750_MOESM3_ESM.docx]

**Description of Additional Supplementary Files**

File Name: Supplementary Dataset 1

Description: Annotated list of all compounds tested in chemogenomic screen. Compounds with sufficient data collected for final analysis are ranked according to their respective impact on BDNF-induced nuclear Arc expression. Compounds excluded from final analysis are listed separately with explanation.
